# The Influence of Carbon Black Colloidal Properties on the Parameters of the Kraus Model

**DOI:** 10.3390/polym15071675

**Published:** 2023-03-28

**Authors:** Kirsty J. Rutherford, Keizo Akutagawa, Julien L. Ramier, Lewis B. Tunnicliffe, James J. C. Busfield

**Affiliations:** 1 School of Engineering and Materials Science, Queen Mary University of London, London E1 4NS, UK; 2 Slb Cambridge Research, Cambridge CB3 0EL, UK; 3 Birla Carbon, Marietta, GA 30062, USA

**Keywords:** carbon black, Payne Effect, fletcher-gent effect, Kraus equation, dynamic strain, natural rubber

## Abstract

The Payne Effect (also known as the Fletcher–Gent Effect) has a fundamental impact on the behavior of filled rubber composites and therefore must be considered during their design. This study investigates the influence of carbon black (CB) surface area and structure on the observed Payne Effect and builds on the existing models of Kraus and Ulmer to explain this phenomenon. Dynamic strain sweeps were carried out on natural rubber (NR) compounds containing eight different grades of CB at equivalent volume fractions. The loss and storage moduli were modeled according to the Kraus and Ulmer equations, using a curve optimization tool in SciPy. Subsequent regression analysis provided strong correlations between the fitting parameters and the CB structure and surface area. Using this regression analysis, this work provides further insight into the physical meaning behind the Kraus and Ulmer models, which are phenomenological in nature.

## 1. Introduction

When subjected to dynamic strains beyond 0.1%, filled elastomer composites exhibit a sigmoidal decrease in storage modulus $\left(G^{\prime }\right)$ and a corresponding peak in loss modulus $\left(G^{\prime \prime }\right)$. This phenomenon is known as the Payne Effect or occasionally referred to as the Fletcher–Gent Effect [1,2,3,4]. The Payne Effect is mainly attributed to the strain-dependent breakdown and reformation of particle-particle contacts, held together by weak van der Waal’s forces [4,5,6]. This breakdown results in irreversible energy dissipation, which has fundamental consequences on the performance of an elastomer composite. For certain applications, including tires, belts, and anti-vibration devices, good fatigue resistance is required, and therefore low levels of energy dissipation are preferred to avoid excess heat build-up. In such instances, a small Payne Effect is desirable. For fracture applications, however, energy dissipation allows for the removal of energy from a propagating crack, thus improving the tear resistance of the elastomer [7]. In these cases, a larger Payne Effect would allow for higher levels of energy dissipation. Most engineering applications require a trade-off between these phenomena; hence, there is motivation to model the Payne Effect.

Several models have been proposed to describe the Payne Effect, including the van de Waale, Tricot, and Gerspacher models, the network junction model, the chain-slippage model, and the links–nodes–blobs model [8,9,10,11,12]. Kraus proposed the first quantitative model based on the breakdown and reformation of assumed van der Waals forces between filler aggregates [13]. This model assumes that the amount of network breakdown per cycle, $R_{b}$, is proportional to the number of existing CB contacts, *N*, as a function of strain amplitude, $f_{b}$:

(1) $$R_{b}=k_{b}Nf_{b}$$

where $k_{b}$ is a constant. The network reformation rate, $R_{m}$ is assumed to be proportional to the number of broken contacts as a function of strain amplitude, $f_{m}$:

(2) $$R_{m}=k_{m}\left(N_{0}-N\right)f_{m}$$

where $N_{0}$ is the number of intact contacts in the network and $k_{m}$ is a constant. At equilibrium, when $R_{b}$ = $R_{m}$, *N* is given as:

(3) $$N=\frac{N_{0}}{1+\frac{k_{b}f_{b}}{k_{m}f_{m}}}\;$$

The excess in storage modulus over that at infinite strain, $G^{\prime }\left(\gamma \right)-G_{\infty }^{\prime }$, is assumed to be proportional to the number of existing particle contacts. Using power laws, $f_{b}$ and $f_{m}$ can be rewritten as:

(4) $$f_{b}=\gamma^{m},f_{m}=\gamma^{-m}$$

where γ is half the peak-to-peak strain amplitude and *m* is a constant. With this in mind, the Kraus model for $G^{\prime }$ can be rewritten as:

(5) $$G^{\prime }\left(\gamma \right)=G_{\infty }^{\prime }+\frac{G_{0}^{\prime }-G_{\infty }^{\prime }}{1+{\left(\gamma /\gamma_{c}\right)}^{2m}}\;$$

where $G_{0}^{\prime }$ is $G^{\prime }$ at zero strain amplitude and $\gamma_{c}$ is at characteristic strain equal to ${\left(k_{m}/k_{b}\right)}^{1/2m}$. The $G_{0}^{\prime }-G_{\infty }^{\prime }$ term, commonly denoted as $\Delta G^{\prime }$, equates to the magnitude of the Payne Effect. According to Kraus, the loss modulus at a given strain, $G^{\prime \prime }\left(\gamma \right)$, in excess over the loss modulus at infinite strain, $G_{\infty }^{\prime \prime }$, arises from the energy dissipation associated with the breakdown and reformation of individual contacts. In other terms:

(6) $$G^{\prime \prime }\left(\gamma \right)-G_{\infty }^{\prime \prime }=C_{1}k_{b}Nf_{b}$$

where $C_{1}$ is a constant. With $f_{b}=\gamma^{m}$, Equation (6) can be differentiated to obtain an expression for the maximum $G^{\prime \prime }$, i.e., $G_{m}^{\prime \prime }$. This can be rearranged to derive the Kraus expression for loss modulus:

(7) $$G^{\prime \prime }\left(\gamma \right)=G_{\infty }^{\prime \prime }+\frac{2\left(G_{m}^{\prime \prime }-G_{\infty }^{\prime \prime }\right){\left(\gamma /\gamma_{c}\right)}^{m}}{1+{\left(\gamma /\gamma_{c}\right)}^{2m}}$$

where $\gamma_{c}$ is the strain at which $G^{\prime \prime }\left(\gamma \right)$ is a maximum. The Kraus model of the storage modulus, given by Equation (5), was found to be applicable at various loadings of CB under different loading conditions, including tension, torsion, and shear [14,15]. The Kraus model for the loss modulus, Equation (7), demonstrated lower success in a study carried out by Ulmer, which reported significant deviations in the low strain regions of the model [16]. Kraus predicted that $G^{\prime \prime }$ reduces to $G_{\infty }^{\prime \prime }$ at zero strain because the extent of the broken network available for reformation also reduces to zero, which is not the case experimentally. Consequently, Ulmer proposed an additional contribution which is assumed to be independent of the CB network, but rather the result of an additional loss contribution associated with the process of polymer network breakdown and reformation. This additional term is proportional to the number of particle contacts:

(8) $$G^{\prime \prime }\left(\gamma \right)-G_{\infty }^{\prime \prime }=+C_{1}k_{b}Nf_{b}+C_{2}N$$

Applying the Kraus strain dependencies of $f_{b}$ and $f_{m}$, the $C_{1}k_{b}Nf_{b}$ term becomes the Kraus expression for $G^{\prime \prime }\left(\gamma \right)-G_{\infty }^{\prime \prime }$ obtained from Equation (7). The additional term, $C_{2}N$, is evaluated by replacing *N* with Equation (3). The second term is a simple, empirical choice of function that decreases exponentially with increasing strain:

(9) $$G^{\prime \prime }\left(\gamma \right)=G_{\infty }^{\prime \prime }+\frac{2\left(\Delta G_{k}^{\prime \prime }\right){\left(\gamma /\gamma_{c}\right)}^{m}}{1+{\left(\gamma /\gamma_{c}\right)}^{2m}}+\Delta G_{2}^{\prime \prime }e^{-\gamma /\gamma_{2}}$$

where $G_{m}^{\prime \prime }-G_{\infty }^{\prime \prime }$ of the Kraus model is called $\Delta G_{k}^{\prime \prime }$, and $C_{2}N_{0}$ is denoted as $\Delta G_{2}^{\prime \prime }$. Equation (9) gives a considerably better description of the loss modulus than the Kraus derived alternative (Equation (7)). There are some disagreements between the estimates of *m* and $\gamma_{c}$, obtained from the Kraus $G^{\prime }\left(\gamma \right)$ model (Equation (5)) and the modified $G^{\prime \prime }\left(\gamma \right)$ (Equation (9)) but this is usually less than ±5%. Ulmer demonstrated that Equations (5) and (9) can successfully model the Payne Effect for various types of rubber including NR, styrene butadiene rubber, nitrile butadiene rubber, and butyl rubber. Furthermore, the model accurately predicted the behavior to within ±2% error for compounds containing between 25 and 50 parts per hundred rubber (phr) of high abrasion furnace CB. The accuracy of these models slightly reduces beyond CB loadings of 80 phr, where the error reported increased to ±5%. The Kraus and modified Kraus equations are limited because they are phenomenological in nature and do not provide any information on how to predict the Payne Effect. Observations dating back to the 1960s have reported that the Payne Effect is heavily influenced by the nature of the reinforcing particles [17,18]. Despite these observations, the current models for predicting this behavior do not consider the basic properties of the filler.

CB represents the most popular filler system for rubber-based applications, its reinforcing potential being largely governed by aggregate morphology and surface area. CB exists as aggregates, formed by the fusion of para-crystalline primary particles. The spatial arrangement of these primary particles is defined as the level of ‘structure’. The structure of CB can be quantified using oil absorption measurements [19,20]. Primary particle size can range between 5 nm and 200 nm and is inversely proportional to the surface area. Surface area is typically measured using gas adsorption techniques [21]. CB manufacturers are able to precisely control the structure and surface area of CB, giving rise to a wide selection of commercially available CBs. In this paper, the term ‘colloidal properties’ refers to the structure and surface area of CB. These parameters, alongside surface activity, define the reinforcing potential of a CB filler [22]. Surface activity is related to the reactivity of the chemical groups present on the CB surface as well as the surface energy distribution [23]. It relates to how well a filler is able to interact with the polymer matrix. This particular feature will not be discussed in this paper because the selected CBs all display broadly similar surface chemistries. Many studies have demonstrated the effect of surface area on polymer-filler interface, inter-aggregate distances, and filler networking [24,25,26]. Meanwhile, the increased structure gives rise to occluded rubber which is screened from globally applied strains, effectively increasing the filler volume fraction [27,28,29]. More recently, researchers performed micro-structural studies on the role of structure and surface area of CB to explain observed phenomenology [30]. Particular studies demonstrated the effects of CB structure and surface area on the Payne Effect, concluding that structure and surface area dominate the low strain behavior, with the effects of surface area diminishing to zero as strain increases [31,32].

This work aims to develop an understanding of how the colloidal properties influence the terms in the existing Kraus and the modified Kraus models (Equations (5) and (9)). The goal of this research is to introduce physical meaning to these phenomenological equations and provide CB selection criteria based on their colloidal properties, to achieve the desired Payne Effect.

## 2. Materials and Methods

### 2.1. Materials

**Figure 1:**
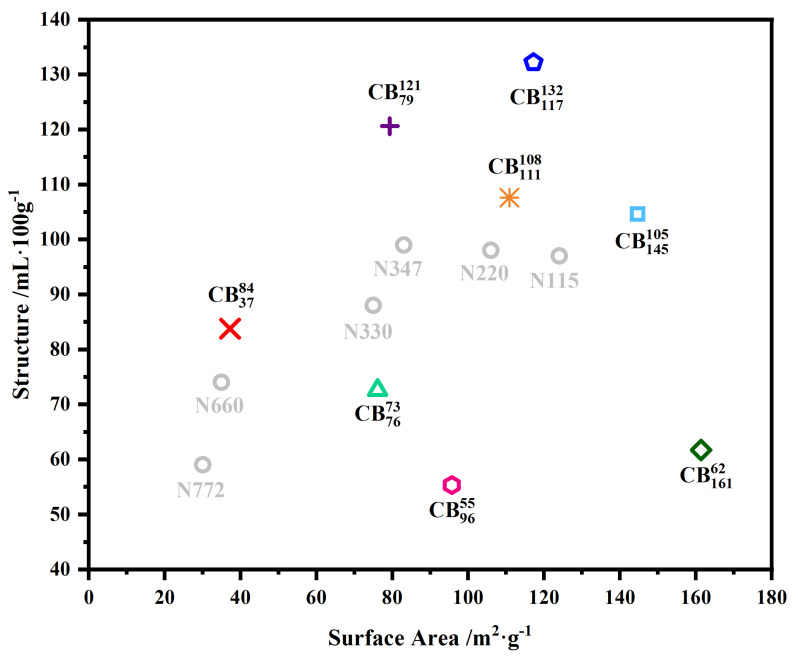
Colloidal plot of CBs, those in black were used during this study. Some conventional CBs have been written in grey for reference. Plot adapted from Kyei-Manu et al. [31].

The data modeled in this study represents a small subset of data collected by Birla Carbon (Marietta, GA, USA). Kyei-Manu et al. proposed a broader study into the effects of CB colloidal properties on the mechanical and dynamic properties of the same materials [31]. Eight grades of CB were selected for this study, based on their relative positions on the colloidal plot, as shown in Figure 1, to cover a broad range of surface areas and structures. Figure 1 also shows some commercially available CBs (N772, N660, N330, N347, N220, and N115). These grades are included in the figure to provide additional context but are not evaluated in this work. The eight selected grades of CB were added to analogous NR-based compounds at equal filler loading of 50 phr. Details of the formulations can be found in Table 1. A naming convention was adopted in this paper to immediately identify the type of CB based on its structure and surface area; details of this are provided in Table 2. The structure (given as compressed oil absorption number, COAN) and surface area (given as statistical thickness surface area, STSA) are denoted as superscript and subscript, respectively. For example, N550 is referred to as CB${}_{37}^{84}$ because it has a structure value of 84 mL·100g${}^{-1}$ and surface area value of 37 m${}^{2}$·g${}^{-1}$. The corresponding rubber compound produced using N550 is referred to using the same naming convention.

Compounding was carried out by Birla Carbon (Marietta, GA, USA) using a 1.6 L capacity Banbury Mixer. A three-stage mixing process was performed, to promote uniform dispersion of the CB. Compounds were vulcanized into sheets measuring 11 mm × 11 mm ×∼2 mm via compression molding. This was carried out at 150 °C for a time of T90 + 5 min, where T90 represents the time taken to reach 90% of the maximum torque on an Alpha Technologies moving die rheometer (MDR) located in Hudson, OH, USA. Interferometric microscopy (IFM) was used to analyze the compound dispersion indices (2–100 μm), following method D in the ASTM standard D2663 [33]. This testing was carried out at Birla Carbon (Marietta, GA, USA).

### 2.2. Dynamic Strain Sweep Characterization

Dynamic strain sweeps, between 0.1% and 39.5% single strain amplitude, were performed at 10 Hz with zero mean strain using an ARES G2 torsional rheometer from TA Instruments, located in New Castle, DE, USA. Testing was carried out at 60 °C. The specimen geometries were disks of 8 mm diameter and approximately 2 mm thickness. Specimens were adhered to the rheometer parallel plate geometry using Loctite 480 adhesive from Henkel, Hemel Hempstead, UK. A compressive pre-force of 100 g was applied to the disks for the duration of the tests. It is noted that with this particular specimen geometry, the strain amplitude varies across the specimen radius; the reported values are therefore the maximum values for the extremity of the disk radius. The samples were pre-conditioned six times at the specified dynamic strain amplitude before collecting the torque-time data. This process was repeated at each strain segment from low to high. Each compound was tested twice.

### 2.3. Curve Fitting and Optimization

The $G^{\prime }\left(\gamma \right)$ data were fitted according to Kraus (Equation (7)) by using a curve optimization tool in SciPy which minimizes the sum of the squared residuals. A full breakdown of the code with added comments can be found in the supplementary information (S1 and S2). An initial optimization was run with a non-negative constraint, meaning that all parameters must take on values equal to or greater than zero. Using the same curve optimization tool, an additional non-negative constraint fitting of the $G^{\prime \prime }\left(\gamma \right)$ data was carried out as per Equation (9). Further constrained optimizations were performed, details of which are discussed in the subsequent Results and Discussion (Section 3.4 and Section 3.5).

## 3. Results and Discussion

### 3.1. IFM Microscopy Results

The Payne Effect is sensitive to the state of filler dispersion, so this feature was important to consider during this work. Compounds with lower levels of filler dispersion are shown to exhibit increased values of $G_{0}^{\prime }$ and decreased values in the median strain required to cause a 50% reduction in $\Delta G^{\prime }$ [34]. The dispersion indices, as determined by IFM, are given in Table 2. Most of the compounds display good levels of dispersion with dispersion indices >98. CB${}_{161}^{62}$ is noted to have a dispersion index of 81.5, which is significantly lower than the other compounds. At a fixed surface area, a decrease in structure leads to an increase in attractive contacts per unit volume of CB; the force necessary to separate aggregates in a pellet, therefore, increases [35]. As a result, grades of CB with a comparatively low structure-to-surface area ratio, for example, CB${}_{161}^{62}$, become increasingly challenging to disperse.

### 3.2. Strain Dependence of $G^{\prime }$ and Non-Negative Curve Optimization

**Figure 2:**
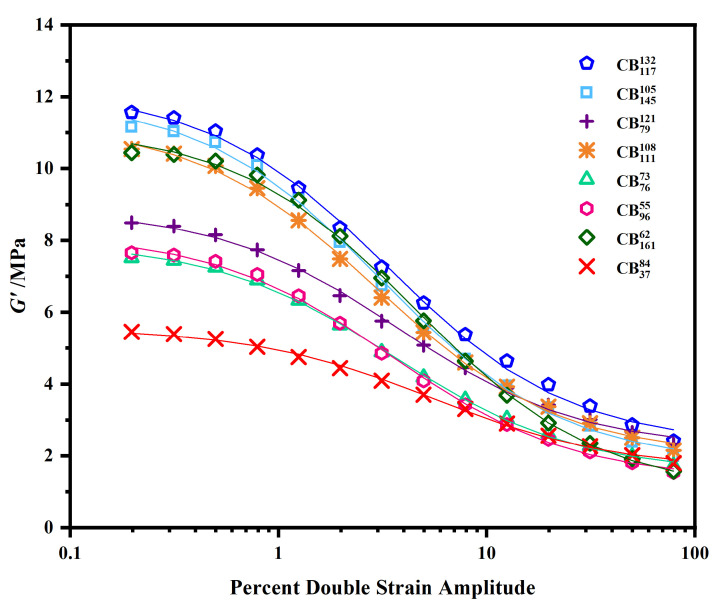
The strain dependence of $G^{\prime }$ for various grades of CB. Symbols represent the experimental data points; lines represent optimized curve fittings.

The raw data for the strain dependence of the storage modulus, $G^{\prime }$, is given in Figure 2. From the first observation, it is clear that the surface area and structure of CB greatly influence the behavior. An initial non-negative curve optimization was performed as per Equation (5), results from which are shown in Table 3. Throughout this paper, fitting parameters are presented as averages across the two repeated measurements; details about individual fitting parameters can be found in the supplementary information (S3–S9). Results across the two independent fittings are in good agreement, proving that there is good agreement between the sets of experimental data. The individual fittings all have $R^{2}$ values greater than 0.997, which indicates that they are all strong fits.

### 3.3. Strain Dependence of G${}^{\prime }$${}^{\prime }$ and Non-Negative Curve Optimization

**Figure 3:**
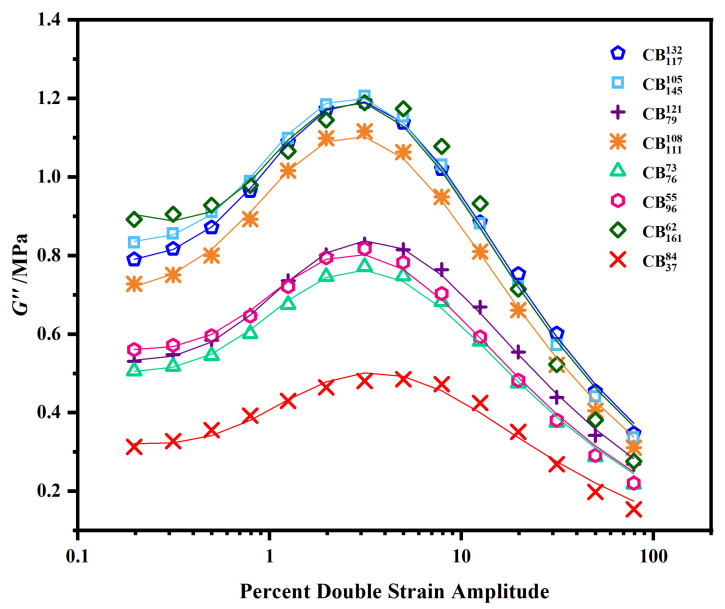
The strain dependence of $G^{\prime \prime }$ for various grades of CB. Symbols represent the experimental data points; lines represent the optimized curve fittings.

The raw data, showing the strain dependence of the loss modulus, $G^{\prime \prime }$, are given in Figure 3. As with the $G^{\prime }$ data, there are obvious differences between the various grades of CB upon initial inspection. The results from the initial unconstrained curve optimizations are given in Table 4. The non-negative constraint meant that values of $G_{\infty }^{\prime \prime }$ across all the fittings were 0, so this information is not included in the table. The $R^{2}$ values for these optimizations are high, with all individual fittings obtaining an $R^{2}$ value of at least 0.996. The repeated data sets are all in good agreement with one another. It is worth noting that values for *m* and $\gamma_{c}$ are different from those found in the previous optimization of $G^{\prime }\left(\gamma \right)$, given in Table 3. By definition, $\gamma_{c}$ should be the strain at which the peak in the loss modulus is located, since this corresponds to the strain at which maximum cluster-cluster breakdown occurs. Unfortunately, the value of $\gamma_{c}$ cannot be taken directly from torsional experiments because this particular geometry shifts the $G^{\prime \prime }\left(\gamma \right)$ peak from its uniform strain position, therefore slight differences in this parameter are expected [16]. The *m* values are shifted to much higher values, the same observation was previously made by Ulmer.

**Figure 4:**
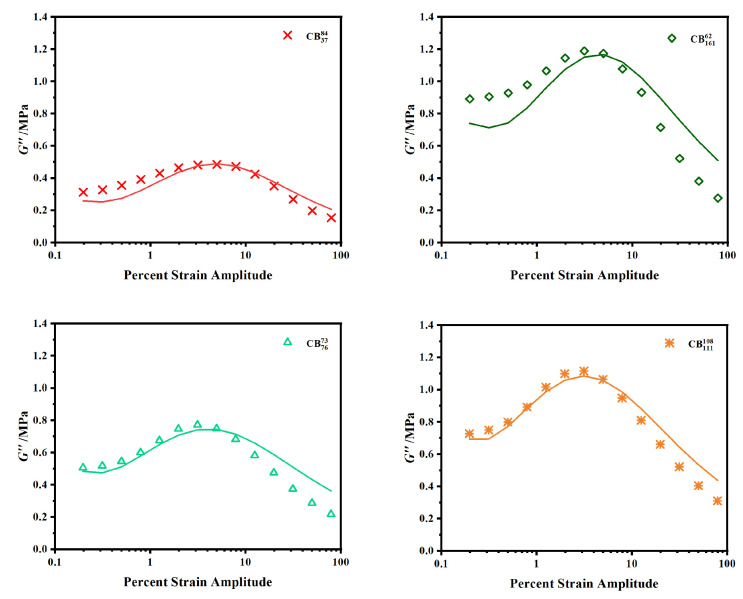
Curve optimizations of $G^{\prime \prime }$ where *m* and $\gamma_{c}$ are constrained as common fitting parameters. Symbols represent experimental data points; lines represent optimized curve fits.

An additional optimization was attempted whereby the $G^{\prime }$ and $G^{\prime \prime }$ optimizations were run simultaneously using $\gamma_{c}$ and *m* as common fitting parameters. The $R^{2}$ coefficients for these curve optimizations are lower than the aforementioned fittings which are particularly reflected in the loss modulus; examples of these fittings are given in Figure 4. A previous evaluation of the Kraus model suggests that the universal value of *m* lies between 0.5 and 0.6, independent of specific filler type [36]. In Table 3, the average *m* value obtained is 0.44 ± 0.4. While these results may only appear to slightly deviate from one another, it was realized that the $\gamma_{c}$ and *m* terms effectively correct for one another, which could add further error to the fittings. For this reason, these initial optimizations were deemed to be inappropriate.

### 3.4. Selection of *m*

In theory, there should be agreement between the *m* and $\gamma_{c}$ values across the models for storage and loss modulus [16]. In practice, there are slight variations between the individual optimizations, as reported in Table 3 and Table 4; this was also recognized by Ulmer when he proposed the modified Kraus model for the loss modulus. The *m* term exhibits a notable strain-dependency that has not been accounted for by either model, which adds a significant complication to its assignment. During torsional shear measurements, specimens do not undergo uniform strain, which adds further complexity to quantifying *m*. In the previous unconstrained optimizations, it was found that *m* and $\gamma_{c}$ are interdependent. This means that any uncertainty in assigning *m* will have direct consequences for $\gamma_{c}$. Therefore, to better understand the effects of CB colloidal properties on $\gamma_{c}$, a simple approach was taken to constrain *m* to a fixed value. The simple empirical choice of m=0.55 was selected because this was the average value obtained across all the unconstrained fittings. This value coincides with the literature, which finds the universal range of *m* to be between 0.5 and 0.6, regardless of filler type [36].

### 3.5. Constrained Curve Optimizations of $G^{\prime }\left(\gamma \right)$ and $G^{\prime \prime }(\gamma$)

Results from the constrained curve optimizations of the storage and loss modulus are given in Table 5 and Table 6. It is worth noting that only the $\gamma_{c}$ parameter was affected by the constraint. Applying the constrained value of *m* reduced the average error in the value of $\gamma_{c}$ across the optimizations for storage and loss modulus from 33.0% to 24.0%. The $R^{2}$ values for the individual optimizations remain strong for the $G^{\prime }\left(\gamma \right)$ data but are slightly reduced for the $G^{\prime \prime }\left(\gamma \right)$ optimizations. In particular, the selected *m* value does not appear to be appropriate for CB${}_{161}^{62}$. The $R^{2}$ value for this CB has been significantly reduced by the *m* constraint. In the initial fitting given in Table 4, this species of CB has the highest *m* value of 0.82. This corresponds to a sharper peak in the loss modulus, which could relate to the lower dispersion index observed in this compound.

The $\gamma_{2}$ term appears to be independent of filler type, showing low variation across all compounds, as seen in Table 6. This parameter was devised by Ulmer as part of an additional term, independent of the filler network, which decreases exponentially with increasing strain. It is, therefore, assumed that this parameter is independent of CB grade and that further optimization of the other fitting parameters could be achieved by constraining this value. For these reasons, an additional curve optimization was performed, assigning $\gamma_{2}$ as 0.23, the average value obtained from Table 6. Results from this optimization are given in Table 7.

This additional constraint has caused minor changes to the other fitting parameters but $R^{2}$ values are largely similar. The optimization of CB${}_{161}^{62}$ results in a far lower $R^{2}$ value compared to the other compounds. This may be related to the lower dispersion index obtained for CB${}_{161}^{62}$, as previously determined in Table 1. The level of filler dispersion is known to affect various aspects of the Payne Effect [34]. Therefore, this particular grade of CB has been excluded from further regression analysis.

### 3.6. Regression Analysis

The Kraus and the modified Kraus equations use a phenomenological approach to successfully model the Payne Effect. The aim of this work is to bring physical meaning to these phenomenological equations. To achieve this, multiple linear regression analysis was performed on the fitting parameters obtained in Table 5 and Table 6. This analysis was carried out in Origin 2019, which bases calculations on Equation (10).

(10) $$Y=C+\beta_{St}\left(COAN\right)+\beta_{SA}\left(STSA\right)+\epsilon$$

where *Y* is the dependent property being examined (in this case the fitting parameters), *C* is the intercept, $\beta_{St}$ is the coefficient of structure, $\beta_{SA}$ is the coefficient of surface area and ϵ is the error. Full regression results are provided in Table 8 and Table 9. Only those values with a corresponding *p*-value < 0.05 are statistically significant. Values that do not meet this requirement have been highlighted in red bold font.

From the storage modulus regression results given in Table 8, a reversal in dominating colloidal property is observed as the strain amplitude is increased. At low strains, indicated at $G_{0}^{\prime }$, both structure and surface area contribute towards the observed modulus value. The magnitude of $\beta_{SA}$ is larger than $\beta_{St}$, which indicates that surface area has a larger effect on $G_{0}^{\prime }$. At higher strains in the region of $G_{\infty }^{\prime }$, the surface area becomes statistically non-relevant and the structure dominates the behavior. At small strains, rigid filler networks augment the storage modulus, with the extent of networking being attributed to the number of aggregates per unit volume, which is related to their surface area. At high strains, these particle networks were broken down and the primary stiffening mechanisms are caused by strain amplification, a product of occluded rubber, which is determined by structural effects, among other factors. Despite subtle differences between $\gamma_{c}$ values determined from the storage and loss modulus optimizations, in both cases, the results are defined by surface area with structure playing a statistically insignificant role. As CB surface area increases, $\gamma_{c}$ values decrease. The negative correlation being observed relates back to the average number of aggregates per unit volume being higher for higher surface area CBs. A subsequent lowering in inter-aggregate distance requires smaller strains to reach critical breakdown.

Results from the regression analysis of $G^{\prime \prime }$ are given in Table 9. The $G_{m}^{\prime \prime }$ parameter refers to the height of the peak in $G^{\prime \prime }\left(\gamma \right)$ which is affected by the surface area and structure of CB, with the surface area having a much larger influence. $G_{m}^{\prime \prime }$ defines the peak in the loss modulus, a product of energy dissipative mechanisms including filler–filler breakdown and polymer-filler slippage phenomena. Statistically, these events have a much higher probability of occurring as the surface area is increased. The $\Delta G_{2}^{\prime \prime }$ term is an additional polymer network term that controls the height of the small strain augmentation in loss modulus. Despite this contribution being polymer-related in definition, the surface area of CB appears to have a strong positive effect on the outcome. During their work, Ulmer investigated several different types of rubber, demonstrating that $\Delta G_{2}^{\prime \prime }$ is dependent on the polymer matrix. As a result, it was concluded that the small strain augmentation is a result of polymer networking. In this work, the polymer remains constant across all eight compounds. The strong correlation between CB surface area and $\Delta G_{2}^{\prime \prime }$ indicates that an additional contribution to the height of the small strain modulus may arise from intact filler networking. As previously discussed, the level of filler networking is dependent on CB surface area, which explains the positive correlation being observed in $\Delta G_{2}^{\prime \prime }$.

## 4. Conclusions

During this work, the Kraus and the modified Kraus equations were able to successfully model the Payne Effect for a variety of NR-based compounds filled with various CBs which differed in their colloidal properties. An additional constrained optimization was carried out whereby the value of *m* was set to 0.55, to overcome issues faced with the interdependence of the *m* and $\gamma_{c}$ parameters. During this optimization, it was noted that CB dispersion affects the sharpness of the peak in the loss modulus and therefore the value of *m*. This meant that the selected *m* value was inappropriate for the $CB_{161}^{62}$, which displayed the lowest compound dispersion as determined by IFM. The $R^{2}$ values obtained across all other optimizations were only slightly reduced following the addition of the *m* constraint. A final constraint on $\gamma_{2}$ was justified because this parameter showed low variation across all compounds. This constraint had a minimal effect on the $R^{2}$ values.

To bring physical meaning to these models, the roles of CB surface area and structure on the various fitting parameters were assessed using multiple regression analysis. As a result of this analysis, the following conclusions can be made:

- At low strains, indicated at $G_{0}^{\prime }$, there is a small structural contribution to the storage modulus arising from polymer occlusion, but the larger influence is that of the surface area which dictates the level of filler networking.
- At higher strain regions of $G_{\infty }^{\prime }$, the surface area becomes statistically insignificant as the filler network is broken down, leaving behind only the effects of polymer occlusion, which is dictated by CB structure.
- As surface area increases, $\gamma_{c}$, the strain at which maximum cluster-cluster breakdown occurs decreases (when *m* is assumed to be constant). This relates to the reduction in the inter-aggregate distance as filler surface area is increased.
- The peak in the loss modulus, $G_{m}^{\prime \prime }$, is affected by surface area and structure but the surface area has the dominating contribution. Energy dissipation arising from filler-filler breakdown and polymer-filler slippage phenomena has a higher probability of occurring as the surface area is increased.
- The additional polymer network term, $\Delta G_{2}^{\prime \prime }$, correlates positively with filler surface area. This can be linked to higher levels of filler networking observed in high surface area CBs which causes augmentation to the small strain modulus.

As such, it is possible to tailor the magnitude of the Payne Effect. For most fatigue applications seeking low heat build-up and therefore a small Payne Effect, low surface area and highly structured CBs are recommended. For fracture applications that require high energy dissipation and a larger Payne Effect, high surface area and low structure CBs would be preferred. It is noted that these recommendations are only suitable for NR-based compounds with 50 phr CB, although similar observations have previously been made for NBR containing 60 phr CB [32]. Future work should be carried out to determine the effects of changing the filler volume fraction and polymer matrix to build upon the results found herein.

## Figures and Tables

**Table 1:** Compound formulation.

Components	Loading/phr	Manufacturer of Component
NR-SMR CV-60	100	Herman Weber & Co.
Various Grades of CB	50	Birla Carbon
Zinc Oxide	5	Akrochem
Stearic Acid	3	PMC Biogenix
Anti-ozonant/Antioxidant	3	Americas International
Micro-Wax	2	Strahl & Pitsch
Sulphur	2.5	R.E. Carroll
TBBS ^1^-75	0.8	Akrochem

^1^ N-Tertiarybutyl-2-benzothiazole sufonamide, 75% assay.

**Table 2:** Naming convention of CBs; colloidal properties; dispersion indices.

CB Reference	Structure (COAN) /mL·100g${}^{-1}$	Surface Area (STSA) /m${}^{2}$·g${}^{-1}$	Compound Dispersion Index
CB${}_{117}^{132}$	132	117	99.3
CB${}_{145}^{105}$	105	145	98.8
CB${}_{79}^{121}$	121	79	98.8
CB${}_{111}^{108}$	108	111	99.4
CB${}_{76}^{73}$	73	76	98.0
CB${}_{96}^{55}$	55	96	90.2
CB${}_{161}^{62}$	62	161	81.5
CB${}_{37}^{84}$	84	37	98.7

**Table 3:** Non-negative fitting of $G^{\prime }\left(\gamma \right)$ according to Equation (5).

CB	$G_{0}^{\prime }$ /MPa	$G_{\infty }^{\prime }$ /MPa	*m*	$\gamma {}_{c}\%$	$R^{2}$
CB${}_{117}^{132}$	12.32	2.29	0.42	4.56	0.998
CB${}_{145}^{105}$	11.90	1.81	0.41	3.96	0.999
CB${}_{79}^{121}$	9.03	2.25	0.44	4.15	0.998
CB${}_{111}^{108}$	11.41	1.97	0.42	4.18	0.999
CB${}_{76}^{73}$	7.62	1.52	0.45	4.34	0.999
CB${}_{96}^{55}$	8.24	1.32	0.44	4.30	0.999
CB${}_{161}^{62}$	11.36	0.97	0.46	5.39	0.999
CB${}_{37}^{84}$	5.38	1.68	0.48	5.72	0.997

**Table 4:** Non-negative fitting of $G^{\prime \prime }\left(\gamma \right)$ according to Equation (9).

CB	$G_{m}^{\prime \prime }$ /MPa	*m*	$\gamma {}_{c}\%$	$\Delta G_{2}^{\prime \prime }$ /MPa	$\gamma_{2}\%$	$R^{2}$
CB${}_{117}^{132}$	1.04	0.68	5.19	0.56	2.96	0.996
CB${}_{145}^{105}$	1.01	0.73	4.91	0.58	2.79	0.998
CB${}_{79}^{121}$	0.85	0.56	3.21	0.43	0.27	0.999
CB${}_{111}^{108}$	1.09	0.58	4.17	0.61	0.24	0.999
CB${}_{76}^{73}$	0.74	0.60	3.37	0.39	0.34	0.999
CB${}_{96}^{55}$	0.83	0.61	3.14	0.40	0.42	0.999
CB${}_{161}^{62}$	1.13	0.82	5.96	0.81	2.38	0.998
CB${}_{37}^{84}$	0.45	0.67	5.78	0.23	2.10	0.998

**Table 5:** Fitting of $G^{\prime }\left(\gamma \right)$ according to Equation (5) where the value of *m* has been assigned as 0.55.

CB	$G_{0}^{\prime }$/MPa	$G_{\infty }^{\prime }$/MPa	$\gamma {}_{c}\%$	$R^{2}$
CB${}_{117}^{132}$	12.32	2.29	3.16	0.998
CB${}_{145}^{105}$	11.90	1.81	2.81	0.999
CB${}_{79}^{121}$	9.03	2.25	3.10	0.998
CB${}_{111}^{108}$	11.41	1.97	2.97	0.999
CB${}_{76}^{73}$	7.62	1.52	3.32	0.999
CB${}_{96}^{55}$	8.24	1.32	3.24	0.999
CB${}_{161}^{62}$	11.36	0.97	4.14	0.999
CB${}_{37}^{84}$	5.38	1.68	4.59	0.997

**Table 6:** Fitting of $G^{\prime \prime }\left(\gamma \right)$ according to Equation (9) where the value of *m* has been assigned as 0.55.

CB	$G_{m}^{\prime \prime }$/MPa	$\gamma_{c}\%$	$\Delta G_{2}^{\prime \prime }$/MPa	$\gamma_{2}\%$	$R^{2}$
CB${}_{117}^{132}$	1.21	2.85	0.65	0.24	0.996
CB${}_{145}^{105}$	1.20	2.67	0.70	0.23	0.997
CB${}_{79}^{121}$	0.86	3.12	0.45	0.22	0.998
CB${}_{111}^{108}$	1.14	2.66	0.68	0.21	0.991
CB${}_{76}^{73}$	0.73	3.06	0.44	0.21	0.994
CB${}_{96}^{55}$	0.82	2.78	0.54	0.21	0.992
CB${}_{161}^{62}$	1.21	2.90	0.80	0.27	0.964
CB${}_{37}^{84}$	0.47	3.48	0.24	0.27	0.988

**Table 7:** Fitting of $G^{\prime \prime }\left(\gamma \right)$ according to Equation (9) where $\gamma_{2}$ has been assigned as 0.23.

CB	$G_{m}^{\prime \prime }$/MPa	$\gamma_{c}\%$	$\Delta G_{2}^{\prime \prime }$/MPa	$R^{2}$
CB${}_{117}^{132}$	1.21	2.84	0.66	0.996
CB${}_{145}^{105}$	1.20	2.67	0.69	0.997
CB${}_{79}^{121}$	0.85	3.12	0.43	0.998
CB${}_{111}^{108}$	1.13	2.82	0.60	0.991
CB${}_{76}^{73}$	0.73	3.07	0.41	0.994
CB${}_{96}^{55}$	0.81	2.80	0.49	0.993
CB${}_{161}^{62}$	1.22	2.85	0.91	0.964
CB${}_{37}^{84}$	0.48	3.45	0.27	0.982

**Table 8:** Regression analysis of $G^{\prime }$ fitting parameters.

Regression Parameter	$G_{0}^{\prime }$/MPa	$G_{\infty }^{\prime }$/MPa	$\gamma {}_{c}$%
C	0.58	0.644	4.71
$\beta_{St}$	$3.42\times 10^{-2}$	$1.37\times 10^{-2}$	**1.06 × 10^4^**
COAN *p*-value	$2.02\times 10^{-2}$	$1.79\times 10^{-4}$	**0.987**
$\beta_{SA}$	$5.85\times 10^{-2}$	**−1.47 × 10^3^**	$-1.46\times 10^{-2}$
STSA *p*-value	$1.28\times 10^{-3}$	**0.145**	$3.68\times 10^{-2}$
Adjusted $R^{2}$	0.95	0.97	0.60

**Table 9:** Regression analysis of $G^{\prime \prime }$ fitting parameters.

Regression Parameter	$G_{m}^{\prime \prime }$/MPa	$\gamma {}_{c}$%	$\Delta G_{2}^{\prime \prime }$/MPa
C	−4.22 × 10${}^{-4}$	3.49	$5.47\times 10^{-2}$
$\beta_{St}$	$3.07\times 10^{-3}$	**2.35 × 10^−3^**	**7.04 × 10^−4^**
COAN *p*-value	$3.48\times 10^{-2}$	**8.20 × 10^−2^**	**0.213**
$\beta_{SA}$	$6.57\times 10^{-3}$	$-7.97\times 10^{-3}$	$4.07\times 10^{-3}$
STSA *p*-value	$3.56\times 10^{-4}$	$5.83\times 10^{-4}$	$4.13\times 10^{-4}$
Adjusted $R^{2}$	0.95	0.94	0.94

## Data Availability

The data presented in this study is available on request from Lewis B. Tunnicliffe.

## Supplementary Materials

The following supporting information can be downloaded at: https: //www.mdpi.com/article/10.3390/polym1010000/s1..
