# Supplementary material for: The Influence of Carbon Black Colloidal Properties on the Parameters of the Kraus Model
Source: Polymers (Basel). 2023 Mar 28;15(7):1675. doi: 10.3390/polym15071675 (PMC10097183; doi:10.3390/polym15071675)
Supplement: Supplementary file 1 [file polymers-15-01675-s001.zip › polymers-2258656-supplementary.pdf]

Supplementary Information

S1: Example code for $G'(\gamma)$

```
import matplotlib.pyplot as plt
import pandas as pd
import numpy as np
from scipy.optimize import curve_fit
data = f'C:/Users/ruthe/OneDrive/Documents/Kraus/N550s1.xlsx'
df = pd.read_excel(data)
x = df['Strain']
y = df['StorageModulus']
p0=[2,35,5,0.55]
#initial guesses for Ginf, Gmax, strainc and m respectively
def Kraus(x, Ginf, Gmax, strainc, m):
    return Ginf +(Gmax-Ginf)/(1+(((x))/(strainc**(2*m))))
popt, pcov = curve_fit(Kraus,x,y,p0,bounds=((0,0,0,0.55),(100,45,10,0.551)))
#parameter optimisation step bounds define the min and max values
Einf = round(popt[0],3)
Emax = round(popt[1],3)
strainc = round(popt[2],3)
mo = round(popt[3],3)
print ('Gmax', Gmax)
print('Ginf=',Ginf)
print('strainc=',strainc)
print('m=',m)

plt.plot(x,y,'ro',label='Experiment')
plt.plot(x,Kraus(x,*popt),'b--',label='BestFitCurve')
plt.grid()
plt.legend(loc='best')
#Calculation R^2
residuals = y-Kraus(x,*popt)
ss_res =np.sum(residuals**2)
ss_tot = np.sum((y-np.mean(y))**2)
r_squared=1-(ss_res/ss_tot)
plt.xscale("log")
print('rsq=',r_squared)

plt.show()
```

S2: Example code for $G''(\gamma)$

```
import matplotlib.pyplot as plt
import pandas as pd
import numpy as np
from scipy.optimize import curve_fit

data = f'C:/Users/ruthe/OneDrive/Documents/Kraus/N550s1loss.xlsx'
df = pd.read_excel(data)
x = df['Strain']
y = df['LossModulus']
p0=[0.0005,5.5,3.93,0.55,2,0.23]
def Kraus(x, Ginf, Gmax, strainc, m, deltaG2, strain2):
    return Ginf +((2*Gmax-((Ginf)))*(x/strainc)**m)/(1+(x/strainc)**(2*m))
    +deltaG2*np.exp((-(x))/strain2)
popt, pcov = curve_fit(Kraus,x,y,p0,bounds=((0,0,0,0.549,0,0.22),
(500,10,10,0.55,10,0.23)))
Einf = round(popt[0],3)
Emax = round(popt[1],3)
mo = round(popt[3],3)
strainc = round(popt[2],3)
deltaE2 = round(popt[4],3)
strain2 = round(popt[5],3)

print ('Gmax =', Gmax)
print('Ginf=',Ginf)
print('strainc=', strainc)
print('m=',m)
print('deltaG2=', deltaG2)
print('strain2=', strain2)

plt.plot(x,y,'ro',label='Experiment')
plt.plot(x,Kraus(x,*popt),'b--',label='BestFitCurve')
plt.grid()
plt.legend(loc='best')
residuals = y-Kraus(x,*popt)
ss_res =np.sum(residuals**2)
ss_tot = np.sum((y-np.mean(y))**2)
r_squared=1-(ss_res/ss_tot)
print(r_squared)
plt.xscale("log")

plt.show()
```

S3: Non-negative constrained individual fitting parameters for $G'(\gamma)$

CB	G'_0 /MPa		G'_∞ /MPa		m		γ_c %		R^2	
CB^{132}_{117}	12.19	12.44	2.33	2.25	0.42	0.42	4.26	4.86	0.998	0.999
CB^{105}_{145}	11.96	11.84	1.80	1.81	0.41	0.41	4.02	3.90	0.999	0.999
CB^{121}_{79}	8.85	9.21	2.22	2.29	0.46	0.43	4.50	3.80	0.998	0.998
CB^{108}_{111}	11.26	11.56	2.01	1.94	0.42	0.42	3.76	4.60	0.999	0.999
CB^{73}_{76}	7.95	7.30	1.55	1.50	0.45	0.45	4.22	4.45	0.999	0.999
CB^{55}_{96}	8.17	8.31	1.34	1.30	0.44	0.45	4.15	4.44	0.999	0.999
CB^{62}_{161}	11.11	11.61	1.02	0.92	0.46	0.47	5.22	5.56	0.999	0.999
CB^{84}_{37}	5.55	5.21	1.64	1.71	0.48	0.48	5.94	5.51	0.998	0.997

S4: Non-negative constrained individual fitting parameters for $G''(\gamma)$

CB	G''_m /MPa		G''_∞ /MPa		m		γ_c %		$\Delta G''_2$		γ_2 %		R^2	
CB^{132}_{117}	1.02	1.07	0.00	0.00	0.66	0.70	5.06	5.33	0.52	0.61	3.18	2.75	0.996	0.996
CB^{105}_{145}	1.01	1.01	0.00	0.00	0.73	0.73	4.92	4.93	0.57	0.58	2.89	2.70	0.998	0.997
CB^{121}_{79}	0.84	0.88	0.00	0.00	0.57	0.56	3.48	2.96	0.39	0.47	0.34	0.21	0.998	1.000
CB^{108}_{111}	1.12	1.06	0.00	0.00	0.58	0.70	2.84	5.50	0.61	0.60	0.25	0.23	0.998	1.000
CB^{73}_{76}	0.78	0.71	0.00	0.00	0.60	0.60	3.27	3.47	0.41	0.37	0.34	0.35	1.000	0.999
CB^{55}_{96}	0.82	0.84	0.00	0.00	0.61	0.61	3.08	3.22	0.33	0.48	0.47	0.37	1.000	0.999
CB^{62}_{161}	1.09	1.17	0.00	0.00	0.81	0.83	5.64	6.29	0.78	0.84	2.28	2.49	0.999	0.998
CB^{84}_{37}	0.47	0.43	0.00	0.00	0.69	0.65	5.92	5.64	0.24	0.21	2.10	2.09	0.998	0.998

S5: Constrained individual fitting parameters for $G'(\gamma)$ where m has been assigned as 0.55

CB	G'_0 /MPa		G'_∞ /MPa		m		γ_c %		R^2	
CB^{132}_{117}	12.19	12.44	2.33	2.25	0.55	0.55	3.00	3.33	0.998	0.999
CB^{105}_{145}	11.96	11.84	1.80	1.81	0.55	0.55	2.84	2.77	0.999	0.999
CB^{121}_{79}	8.85	9.21	2.22	2.29	0.55	0.55	3.37	2.84	0.998	0.998
CB^{108}_{111}	11.26	11.56	2.01	1.94	0.55	0.55	2.72	3.21	0.999	0.999
CB^{73}_{76}	7.95	7.30	1.55	1.50	0.55	0.55	3.21	3.45	0.999	0.999
CB^{55}_{96}	8.17	8.31	1.34	1.30	0.55	0.55	3.14	3.35	0.999	0.999
CB^{62}_{161}	11.11	11.61	1.02	0.92	0.55	0.55	3.99	4.28	0.999	0.999
CB^{84}_{37}	5.55	5.21	1.64	1.71	0.55	0.55	4.75	4.44	0.998	0.997

S6: Constrained individual fitting parameters for $G''(\gamma)$ where m has been assigned as 0.55

CB	G''_m /MPa		G''_∞ /MPa		m		γ_c %		$\Delta G''_2$		γ_2 %		R^2	
CB^{132}_{117}	1.19	1.22	0.00	0.00	0.55	0.55	2.82	2.87	0.64	0.65	0.23	0.25	0.998	0.995
CB^{105}_{145}	1.20	1.19	0.00	0.00	0.55	0.55	2.68	2.65	0.68	0.71	0.23	0.22	0.998	0.997
CB^{121}_{79}	0.84	0.87	0.00	0.00	0.55	0.55	3.31	2.92	0.42	0.49	0.25	0.19	0.997	0.999
CB^{108}_{111}	1.11	1.16	0.00	0.00	0.55	0.55	2.95	3.16	0.47	0.41	0.20	0.21	0.994	0.994
CB^{73}_{76}	0.76	0.70	0.00	0.00	0.55	0.55	2.95	3.16	0.47	0.41	0.20	0.22	0.994	0.994
CB^{55}_{96}	0.81	0.83	0.00	0.00	0.55	0.55	2.75	2.82	0.53	0.54	0.21	0.21	0.994	0.994
CB^{62}_{161}	1.19	1.24	0.00	0.00	0.55	0.55	2.76	3.03	0.79	0.81	0.26	0.28	0.973	0.957
CB^{84}_{37}	0.50	0.45	0.00	0.00	0.55	0.55	3.54	3.43	0.25	0.22	0.27	0.27	0.983	0.993

S7: Constrained individual fitting parameters for $G''(\gamma)$ where m has been assigned as 0.55 and γ_2 has been assigned as 0.23

CB	G''_m /MPa		G''_∞ /MPa		m		γ_c %		$\Delta G''_2$		γ_2 %		R^2	
CB^{132}_{117}	1.19	1.22	0.00	0.00	0.55	0.55	2.83	2.86	0.63	0.69	0.23	0.23	0.998	0.995
CB^{105}_{145}	1.20	1.19	0.00	0.00	0.55	0.55	2.68	2.57	0.69	0.69	0.23	0.23	0.998	0.997
CB^{121}_{79}	0.84	0.87	0.00	0.00	0.55	0.55	3.29	2.95	0.45	0.41	0.23	0.23	0.997	0.999
CB^{108}_{111}	1.11	1.16	0.00	0.00	0.55	0.55	2.71	2.94	0.42	0.40	0.23	0.23	0.994	0.985
CB^{73}_{76}	0.76	0.69	0.00	0.00	0.55	0.55	2.98	3.17	0.42	0.40	0.23	0.23	0.994	0.994
CB^{55}_{96}	0.80	0.83	0.00	0.00	0.55	0.55	2.77	2.84	0.48	0.50	0.23	0.23	0.994	0.992
CB^{62}_{161}	1.19	1.25	0.00	0.00	0.55	0.55	2.72	2.97	0.88	0.95	0.23	0.23	0.973	0.956
CB^{84}_{37}	0.50	0.45	0.00	0.00	0.55	0.55	3.49	3.40	0.29	0.25	0.23	0.23	0.978	0.986

S8: Table of standard deviations for optimized final fittings of $G'(\gamma)$

CB	G'_0 /MPa	G'_∞ /MPa	m	γ_c %
CB^{132}_{117}	0.13	0.04	0.00	0.17
CB^{105}_{145}	0.06	0.01	0.00	0.03
CB^{121}_{79}	0.18	0.04	0.00	0.27
CB^{108}_{111}	0.15	0.04	0.00	0.25
CB^{73}_{76}	0.33	0.02	0.00	0.11
CB^{55}_{96}	0.07	0.02	0.00	0.11
CB^{62}_{161}	0.25	0.05	0.00	0.15
CB^{84}_{37}	0.17	0.03	0.00	0.16

S9: Table of standard deviations for optimized final fittings of $G''(\gamma)$

CB	G''_m /MPa	G''_∞ /MPa	m	γ_c %	$\Delta G''_2$	γ_2 %
CB^{132}_{117}	0.01	0.00	0.00	0.02	0.03	0.00
CB^{105}_{145}	0.01	0.00	0.00	0.05	0.00	0.00
CB^{121}_{79}	0.02	0.00	0.00	0.17	0.02	0.00
CB^{108}_{111}	0.03	0.00	0.00	0.11	0.07	0.00
CB^{73}_{76}	0.04	0.00	0.00	0.10	0.01	0.00
CB^{55}_{96}	0.01	0.00	0.00	0.03	0.01	0.00
CB^{62}_{161}	0.03	0.00	0.00	0.12	0.04	0.00
CB^{84}_{37}	0.02	0.00	0.00	0.04	0.02	0.00
